# Association between the C-reactive protein-albumin-lymphocyte (CALLY) index and mortality in elderly patients with dysphagia requiring nutritional support

**DOI:** 10.3389/fneur.2025.1650795

**Published:** 2025-09-30

**Authors:** Hui Shen, Tonghu Jin, Qing Mei, Hao Guan, Kuang Yan, Aihua Liu

**Affiliations:** ^1^Cerebrovascular Disease Department, Neurological Disease Center, Beijing Anzhen Hospital, Capital Medical University, Beijing, China; ^2^Beijing Neurosurgical Institute, Beijing Tiantan Hospital, Capital Medical University, Beijing, China; ^3^Department of Neurology, Beijing Pinggu Hospital, Beijing, China; ^4^People's Hospital of Ningxia Hui Autonomous Region, Ningxia Medical University, Yinchuan, China

**Keywords:** CALLY index, dysphagia, percutaneous endoscopic gastrostomy, total parenteral nutrition, prognosis

## Abstract

**Background:**

While the novel C-reactive protein-albumin-lymphocyte (CALLY) index—integrating inflammatory, nutritional, and immune markers—has proven valuable for prognosis in various diseases, its utility in predicting outcomes for dysphagia patients is still unclear.

**Methods:**

This retrospective cohort analysis utilized data sourced from the Dryad Digital Repository. The CALLY index (albumin × lymphocyte count/C-reactive protein × 104) was calculated. After logarithmic transformation (Ln-CALLY), the patients were divided into low Ln-CALLY group (≤ 2.21) and high Ln-CALLY group (>2.21). All-cause mortality served as the primary endpoint. The association between Ln-CALLY and outcomes was evaluated using Kaplan-Meier analysis with log-rank testing and multivariable Cox proportional hazards regression. The dose-response relationship was evaluated by restricted cubic splines (RCS).

**Results:**

This study enrolled 253 elderly dysphagia patients. Analysis revealed Ln-CALLY as a significant predictor of all-cause mortality.

**Conclusion:**

The CALLY index shows promise as a predictor of mortality risk and long-term outcomes in older dysphagia patients.

## Introduction

Dysphagia is a clinical manifestation arising from numerous underlying diseases (1), and the prevalence of dysphagia varies between 6% and 50% for different age groups (2), ranging from 11% to 16% for oropharyngeal dysphagia in the non-hospitalized older population to 55% for older adults who are unwell (3). Dysphagia is very common in the elderly and in people with conditions such as stroke (4, 5); Dysphagia has been reported in more than 70% of patients with stroke (4). Dysphagia is associated with a doubled hospital length of stay and an increased risk of aspiration pneumonia (6); Aspiration, chest infection, dehydration, and even death are among the common complications associated with dysphagia (2). Furthermore, dysphagia can contribute to malnutrition in patients, which in turn affects their rehabilitation prognosis (7). At present, the prognosis assessment of elderly patients with dysphagia who need nutritional support is not sufficient, so it is important to find effective biomarkers.

At present, it is believed that as a sensitive index, Lymphocyte levels serve as an indicator of both immune and nutritional status in older adults (8, 9). A retrospective study found that elevated C-reactive protein/albumin ratio correlates with increased mortality among older Japanese patients with dysphagia (10). The CALLY index, calculated from C-reactive protein, serum albumin, and lymphocyte levels, provides a novel composite measure of nutritional and inflammatory status. The CALLY index is derived using the formula: (albumin × lymphocyte)/(CRP × 10^4^) (11). A number of previous studies demonstrate that the CALLY index outperforms conventional inflammatory markers in predicting prognosis for cancer, cardiorenal syndrome, and sarcopenia patients (12–14).

This study determines the association of the CALLY index with mortality in older dysphagia patients requiring nutritional support, aiming to identify superior prognostic biomarkers.

## Materials and methods

### Data source

This secondary analysis examined older dysphagia patients receiving percutaneous endoscopic gastrostomy (PEG) or total parenteral nutrition (TPN) at a Japanese center (2014–2017). Study data were sourced from Dryad Digital Repository (doi: 10.5061/dryad.gg407h1) Miyanomori Memorial Hospital's Ethics Board waived informed consent and approved resource utilization, as the study utilized publicly available and anonymized data, obviating the need for individual patient consent.

### Criteria for population selection

Nutritional support modality (PEG/TPN) was determined through clinician-patient/family consensus based on clinical evaluation. Exclusion criteria comprised: (1) advanced cancer; (2) PEG for gastric decompression; (3) pre-2014 PEG placement.

### Variables

The study encompassed four main components: (1) Demographic variables: age and gender; (2) Nutritional variables: clinical frailty scale (CFS), body mass index (BMI), and nutritional support (NS) type, nutrient intake; (3) Comorbidities: cardiovascular diseases (CVD), neuromuscular diseases (NMD), ischemic heart diseases (IHD), chronic heart failure (CHF), chronic pulmonary diseases (CPD), chronic liver diseases (CLD), and chronic kidney diseases (CKD); (4) laboratory biomarkers measured within 7 days prior to nutritional support initiation: albumin (ALB), total lymphocyte count (TLC), C-reactive protein (CRP), total cholesterol (TC), and hemoglobin (Hb).

### C-reactive protein-albumin-lymphocyte

**Figure 1:**
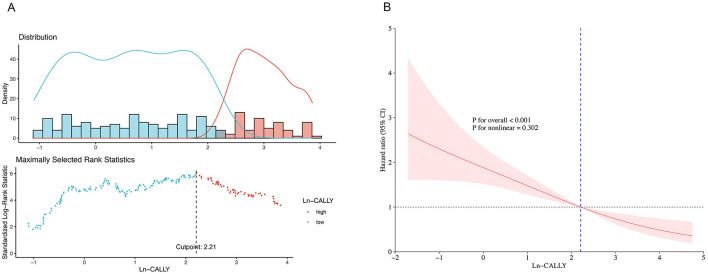
Maximally selected log-rank statistics for cutoff point of LN-CALLY **(A)** and restricted cubic spline curve **(B)** of the LN-CALLY and all-cause mortality.

The CALLY index was determined using the following formula, adapted from prior research: CALLY index = [albumin level (g/dl) × lymphocyte count (cells/μl)]/[CRP level (mg/dl) × 10^4^]. Given its right-skewed distribution (Supplementary Figure 1), CALLY values underwent log transformation (Ln-CALLY) for regression analysis. Patients were stratified into two groups based on Ln-CALLY values using maximally selected rank statistics (Figure 1A): a low group (Ln-CALLY ≤ 2.21) and a high group (Ln-CALLY > 2.21).

### Clinical outcomes

The primary outcome was all-cause mortality, defined as the time from initiation of nutritional support to death, irrespective of etiology. The secondary outcomes were pneumonia-cause mortality, defined as the duration from the onset of nutritional support to pneumonia-associated death, oral recovery, discharge to home, the incidence of severe pneumonia and sepsis.

### Statistical analysis

Patient characteristics are summarized overall and by Ln-CALLY (low vs. high). Continuous variables are reported as medians [interquartile ranges (IQRs)], and categorical variables as frequencies (%). Continuous data were analyzed using the Wilcoxon rank-sum test. Categorical data were analyzed using Pearson's chi-square test or Fisher's exact test, as appropriate. To ensure data completeness and increase the accuracy of results, multiple imputation methods were used to handle missing values using a chained equation approach method and 5 replications to address the missing data (“mice” package).

Restricted cubic splines (knots at 10th, 50th, and 90th percentiles; reference, Ln-CALLY = 2.21) explored the dose–response relationship between Ln-CALLY and all-cause mortality, with non-linearity tested by the likelihood-ratio test (*P* for non-linear). The log-rank test and Kaplan–Meier survival curves were used to compare all-cause and pneumonia-related mortality between the two Ln-CALLY groups. To assess Ln-CALLY's independent predictive value, we established multivariate Cox proportional hazards regression models with three models for confounder adjustment. Model 1 was adjusted for age and gender. Model 2 was adjusted for age, gender, NS, BMI, CFS and nutrient intake. Model 3 incorporated all Model 2 variables plus severe dementia, CLD, CKD and TC. Schoenfeld residuals confirmed the proportional-hazards assumption for every covariate (all *P* > 0.05); any future violations will be addressed with time-varying covariates or stratified analyses.

To assess the robustness of our findings, we conducted sensitivity analyses using the median and tertiles of the Ln-CALLY cut-off, and various imputation methods including predictive mean matching (PMM), random-forest imputation, complete-case analysis, single-stochastic normal imputation, and classification and regression threes (CART). Additionally, we performed direct comparisons of CALLY's predictive performance (e.g., ROC/AUC) against the prognostic nutritional index (PNI).

Stratification and interaction analyses were also performed to evaluate the effects of variables such as age (<85 and ≥85 years), gender, NS (PEG and TPN), severe dementia, CVD, NMD, CHF, CLD and CKD. Forest plots were used to present the results. A two-tailed test with *P* < 0.05 was considered statistically significant. All statistical analyses were conducted using R (version 4.4.3).

## Results

### Participant

Our analysis encompassed 253 elderly patients with dysphagia, of whom 154 (61.0%) were female. The patients had a median age 85.0 years (IQR: 79.0–89.0) and BMI 19.1 kg/m^2^ (IQR: 17.1–20.8). Their median nutrient intake was 900.0 Kcal/day (IQR: 892.0–930.0). Of the 253 patients, 180 underwent PEG feeding and 73 received TPN. The all-cause and pneumonia-related mortality rates were 55% and 17%, respectively. Oral recovery and sepsis occurred in 5.9% and 15% of patients, severe pneumonia in 5.9%, and 15% were discharged home.

The median Ln-CALLY values were 0.4 (IQR: −0.2–2.8) for the low group and 3.3 (IQR: 2.7–4.2) for the high group. Median Ln-CALLY values were 0.4 (IQR −0.2–2.8) in the low group vs. 3.3 (IQR 2.7–4.2) in the high group. Baseline characteristics (Table 1) were analyzed across the Ln-CALLY groups. High Ln-CALLY group patients demonstrated a greater likelihood of PEG utilization, increased daily nutrient intake, and a higher prevalence of CVD. Conversely, this group exhibited a lower prevalence of severe dementia and CHF. Significant disparities were noted between the groups in terms of ALB, TLC count, CRP, TC, CCI, and Hb levels (all *P* < 0.05). Relative to the low group, high-group patients demonstrated significantly reduced rates of severe pneumonia, sepsis, all-cause mortality, and pneumonia-related mortality, alongside higher rates of oral intake recovery and home discharge.

**Table 1:** Clinical characteristics of dysphagia patients requiring nutritional support.

**Characteristic**	**Overall *N* = 253^1^**	**Ln-CALLY**		***P*-value^2^**
		**Low** ***N*** = **167**^1^	**High** ***N*** = **86**^1^	
Ln-CALLY	1.4 (−0.2–2.8)	0.4 (−0.7–1.3)	3.3 (2.7–4.2)	<0.001
CALLY	3.9 (0.8–16.8)	1.5 (0.5–3.8)	27.0 (15.6–69.2)	<0.001
**Demographic**				
Age, years	85.0 (79.0–89.0)	85.0 (81.0–90.0)	84.0 (74.0–89.0)	0.060
Gender (Female), *n* (%)	154 (61)	96 (57)	58 (67)	0.12
**Nutritional parameters**				
CFS	8.0 (8.0–8.0)	8.0 (8.0–8.0)	8.0 (8.0–8.0)	0.25
BMI, kg/m^2^	19.1 (17.1–20.8)	18.8 (16.9–20.8)	19.5 (17.5–20.9)	0.19
NS, *n* (%)				<0.001
PEG	180 (71)	104 (62)	76 (88)	
TPN	73 (29)	63 (38)	10 (12)	
Nutrient intake, Kcal/day	900.0 (892.0–930.0)	900.0 (770.0–900.0)	900.0 (900.0–1,200.0)	<0.001
**Comorbidities**				
CVD, *n* (%)	133 (53)	78 (47)	55 (64)	0.009
Severe dementia, *n* (%)	102 (40)	79 (47)	23 (27)	0.002
NMD, *n* (%)	14 (5.5)	6 (3.6)	8 (9.3)	0.080
IHD, *n* (%)	47 (19)	36 (22)	11 (13)	0.089
CHF, *n* (%)	107 (42)	83 (50)	24 (28)	<0.001
CPD, *n* (%)	19 (7.5)	16 (9.6)	3 (3.5)	0.082
CLD, *n* (%)	15 (5.9)	12 (7.2)	3 (3.5)	0.24
CKD, *n* (%)	53 (21)	40 (24)	13 (15)	0.10
**Lab biomarkers**				
ALB, g/dl	3.1 (2.8–3.6)	2.9 (2.6–3.2)	3.6 (3.3–3.8)	<0.001
TLC, mm^3^	1,176.0 (864.0–1,627.0)	1,054.5 (748.8–1,386.0)	1,513.8 (1,167.9–2,046.0)	<0.001
CRP, mg/dl	1.0 (0.3–3.3)	2.3 (1.0–5.1)	0.2 (0.1–0.3)	<0.001
TC, mg/dl	156.0 (128.0–186.0)	148.0 (117.0–175.0)	177.5 (149.0–202.0)	<0.001
Hb, g/dl	11.1 (9.7–12.4)	10.5 (9.1–11.8)	12.3 (10.9–13.4)	<0.001
**Outcomes**				
Severe pneumonia, *n* (%)	69 (27)	53 (32)	16 (19)	0.026
Sepsis, *n* (%)	30 (12)	25 (15)	5 (5.8)	0.033
Oral recovery, *n* (%)	15 (5.9)	5 (3.0)	10 (12)	0.006
Discharge to home, *n* (%)	38 (15)	22 (13)	16 (19)	0.25
All-cause mortality, *n* (%)	138 (55)	113 (68)	25 (29)	<0.001
Pneumonia-cause mortality, *n* (%)	44 (17)	35 (21)	9 (10)	0.037

^1^*n* (%); Median (IQR).

^2^Pearson's Chi-squared test; Wilcoxon rank sum test; Fisher's exact test.

CALLY, C-reactive protein-albumin-lymphocyte index; CFS, clinical frailty scale; BMI, body mass index; NS, nutritional support; CVD, cardiovascular diseases; NMD, neuromuscular diseases; IHD, ischemic heart diseases; CHF, chronic heart failure; CPD, chronic pulmonary diseases; CLD, chronic liver diseases; CKD, chronic kidney diseases; ALB, albumin; TLC, total lymphocyte count; CRP, C-reactive protein; TC, Total cholesterol; Hb, hemoglobin.

### Clinical outcomes

Univariate analysis demonstrated reduced severe pneumonia/sepsis rates and improved oral recovery/home discharge rates in high vs. low groups.

**Figure 2:**
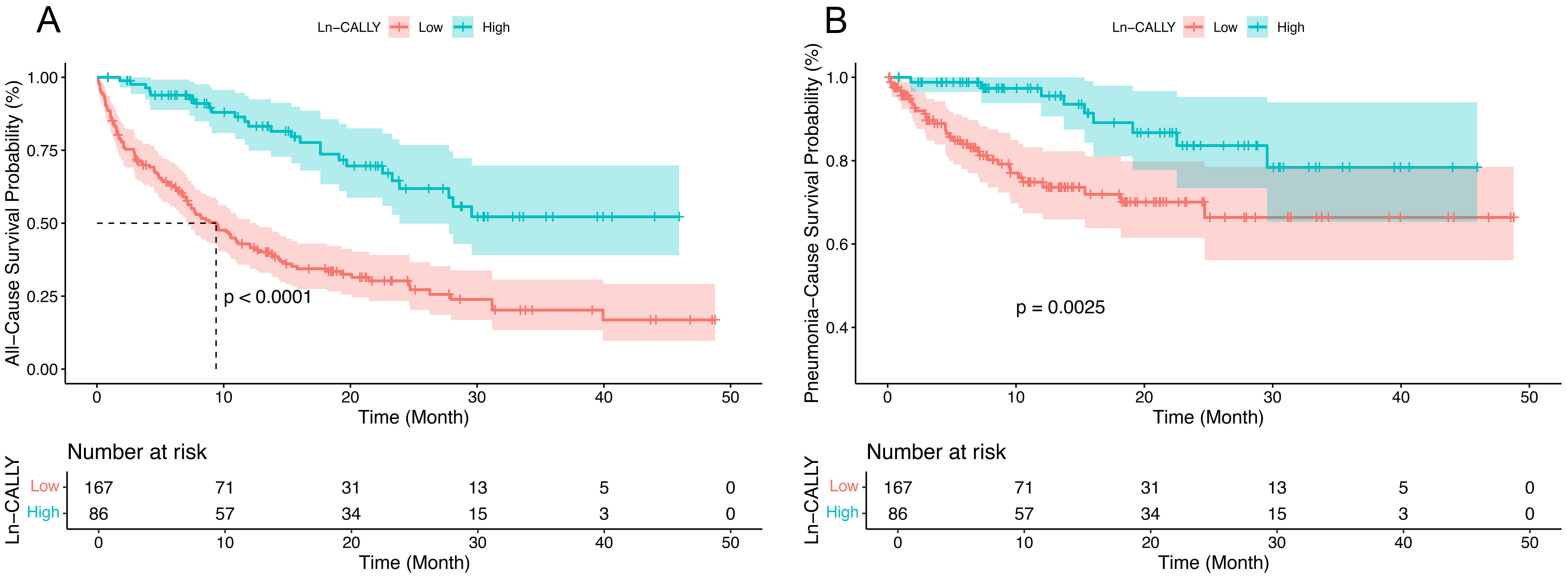
Kaplan–Meier survival analysis curves for all-cause **(A)** and pneumonia-cause survival **(B)**.

An RCS regression model with four knots and a cutoff of Ln-CALLY = 2.21 (reference) revealed a linear association between Ln-CALLY and all-cause mortality (*P* for overall <0.001, *P* for non-linear = 0.302) (Figure 1B). Kaplan-Meier analysis showed significant differences in all-cause (*P* < 0.001) and pneumonia-cause mortality (*P* = 0.0025) across Ln-CALLY groups (Figure 2).

Cox proportional hazards regression models (Table 2) indicated Ln-CALLY was a significant all-cause mortality predictor in **Model 1** (HR: 0.77; 95% CI: 0.70–0.85; *P* < 0.001), **Model 2** (HR: 0.79; 95% CI: 0.72–0.88; *P* < 0.001), and **Model 3** (HR: 0.84; 95% CI: 0.76–0.94; *P* = 0.002) as a continuous variable, with comparable results for pneumonia-cause mortality (all *P* < 0.01).

**Table 2:** Cox proportional hazards models for Ln-CALLY in association with all-cause and pneumonia-cause mortality.

**Outcomes**	**Model 1**		**Model 2**		**Model 3**	
	**HR (95% CI)**	***P*** **-value**	**HR (95% CI)**	***P*** **-value**	**HR (95% CI)**	***P*** **-value**
**All-cause mortality**						
Continuous variable	0.77 (0.70–0.85)	<0.001	0.79 (0.72–0.88)	<0.001	0.84 (0.76–0.94)	0.002
**Categorical**				
Low	Reference		Reference		Reference	
High	0.34 (0.22–0.53)	<0.001	0.43 (0.27–0.68)	<0.001	0.46 (0.29–0.72)	<0.001
**Pneumonia-cause mortality**						
Continuous variable	0.75 (0.63–0.89)	0.001	0.76 (0.64–0.91)	0.003	0.77 (0.64–0.94)	0.009
**Categorical**						
Low	Reference		Reference		Reference	
High	0.39 (0.18–0.82)	0.013	0.39 (0.18–0.84)	0.016	0.44 (0.20–0.97)	0.041

HR, hazard ratio; CI, confidence interval.

Model 1: adjusted age, gender; Model 2: adjusted age, gender, NS, BMI, CFS and Nutrient intake; Model 3: adjusted age, gender, NS, BMI, CFS, Nutrient intake, Severe dementia, CLD, CKD, TC.

As a categorical variable, high Ln-CALLY showed significantly lower all-cause mortality vs. low group. This finding persisted across Cox models: **Model 1** (HR: 0.34; 95% CI: 0.22–0.53; *P* < 0.001), **Model 2** (HR: 0.43; 95% CI: 0.27–0.68; *P* < 0.001), and **Model 3** (HR: 0.46; 95% CI: 0.29–0.72; *P* < 0.001). Multivariate Cox regression analyses for pneumonia-cause mortality showed a similar trend (*P* < 0.05).

### Sensitivity analysis

To further validate our findings, we conducted sensitivity analyses. We recalculated the primary endpoint using the median and upper/lower tertiles in addition to the data-driven threshold identified by maximally selected rank statistics. The hazard ratios remained nearly identical across all cut-points (Supplementary Figure 3), indicating that the observed effects were robust and not dependent on the specific threshold chosen.

We also refitted the multivariable Cox model under five different scenarios for handling missing data: predictive mean matching, random-forest imputation, complete-case analysis, single-stochastic normal imputation, and CART. The point estimates and 95% confidence intervals showed substantial overlap (Supplementary Figure 4), confirming that our conclusions were consistent and insensitive to the underlying missing data mechanism.

### Predictive accuracy

To assess the predictive accuracy of Ln-CALLY and PNI for all-cause mortality at the 2-year timepoint, we conducted ROC curve analyses. The area under the curve (AUC) was 0.689 (95% CI: 0.597–0.782) for Ln-CALLY and 0.684 (95% CI: 0.598–0.771) for PNI, demonstrating very similar performance between the two markers.

### Subgroup analysis

**Figure 3:**
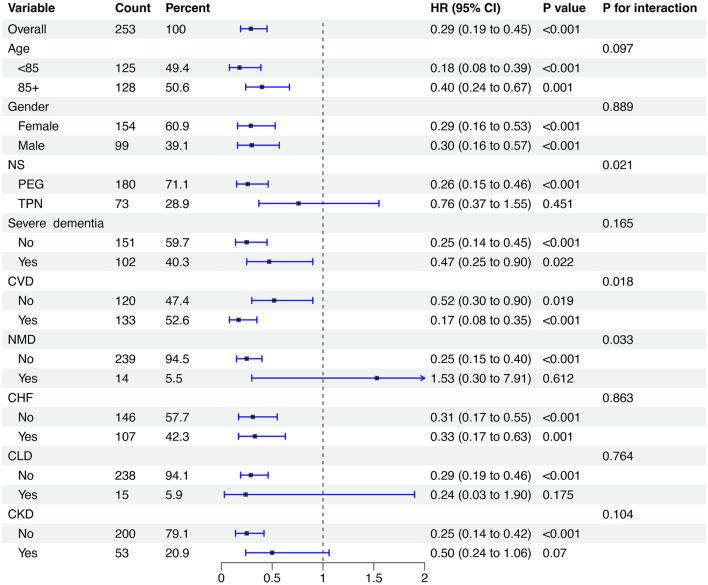
Subgroup analysis of the relationship between LN-CALLY and all-cause mortality. CALLY, C-reactive protein-albumin-lymphocyte index.

To further explore the relationship between Ln-CALLY and all-cause mortality, stratified analyses were conducted based on age (<85 years or ≥85 years), gender, NS, presence of severe dementia, CVD, NMD, CHF, CLD, and CKD (Figure 3). Compared to patients in the low group, only those in the high group who underwent TPN or had severe dementia, NMD, CLD, or CKD showed no significant difference in all-cause mortality (all *P* > 0.05). Notably, significant interactions were observed between Ln-CALLY levels and three clinical parameters: NS (*P* for interaction = 0.021), CVD (*P* for interaction = 0.018), and NMD (*P* for interaction = 0.033).

**Figure 4:**
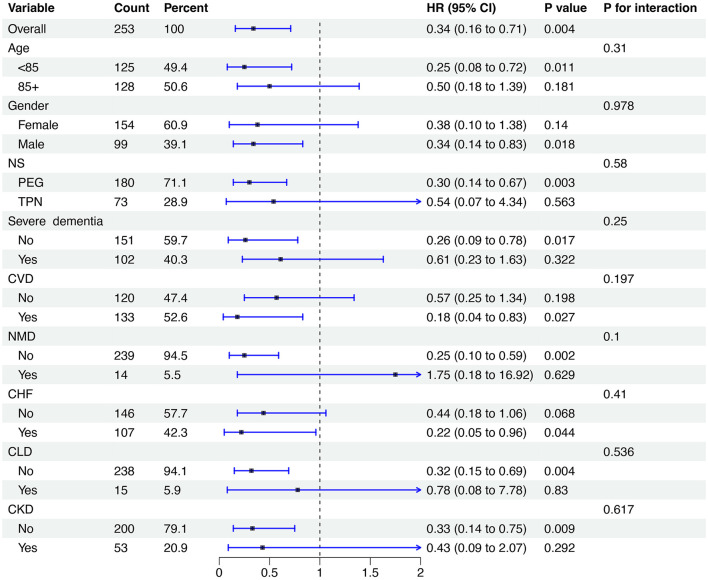
Subgroup analysis of the relationship between LN-CALLY and pneumonia-cause mortality. CALLY, C-reactive protein-albumin-lymphocyte index.

In the subgroup analysis of pneumonia-cause mortality, patients in the high group showed a significantly lower mortality risk than those in the low group in specific subgroups: those aged <85 years, males, those not receiving TPN, and those without severe dementia, CLD, or CKD, as well as those with CVD and CHF (all *P* < 0.05). However, no significant interaction effects were found across these subgroups (*P* for interaction > 0.05) (Figure 4).

## Discussion

This study demonstrated that the CALLY index—a composite measure combining nutritional, inflammatory, and immune status—serves as an independent predictor of mortality and clinical outcomes in older adults with dysphagia. A high level of Ln-CALLY can independently predict a lower risk of death, fewer severe complications and better rehabilitation outcomes, and its protective effect is particularly prominent in certain clinical phenotypes (such as non-TPN-dependent, no severe organ failure). Nutritional status, cardiovascular and neuromuscular diseases may affect the prognostic value of this index.

Dysphagia may be associated with an inflammatory response. When inflammation occurs, skeletal muscle protein catabolism is enhanced, which can further lead to swallowing muscle weakness (15, 16). A study has shown a link between aspiration pneumonia and swallowing muscle atrophy in mouse models (17), and a subsequent clinical retrospective study has also confirmed this association and pointed out that inflammation can affect the prognosis of patients with dysphagia (18). In addition, Nutritional status significantly influences the prognosis of patients with dysphagia. Malnutrition induced by dysphagia can exacerbate dysphagia, forming a vicious cycle that ultimately leads to poor prognosis (19). It is worth noting that there is a complex interaction among inflammation, malnutrition and immune function. Malnutrition impairs immune cell function, compromising the body's capacity to combat infections and regulate inflammation, thereby undermining immune system integrity (20); at the same time, malnutrition exacerbates oxidative stress and inflammatory mediator production, triggering an excessive inflammatory response that worsens both dysphagia severity and its underlying conditions (21).

The CALLY index was originally developed as an immunonutritional assessment tool for hepatocellular carcinoma (HCC) patients. This convenient and concise composite index demonstrates a significant association with HCC patient prognosis. Subsequent research links the CALLY index to prognosis across multiple diseases such as stroke, myocardial infarction, ulcerative colitis and so on (22–26). CALLY index variations are underpinned by inflammation, immune function, and albumin levels, with complex interplay between these factors.

Prior research identified serum albumin <3.5 g/L as an independent predictor of suboptimal clinical outcomes in older adults with dysphagia (27), and based on this, PNI is proposed to predict the mortality of elderly patients with dysphagia (28). In contrast, the CALLY index incorporates CRP measures reflecting systemic inflammation on the basis of integrating nutritional and immune status. This inclusion of the inflammatory dimension, which enables a more comprehensive capture of common pathophysiological conditions in patients, may theoretically provide superior prognostic predictive power over PNI (29).

The CALLY index (CRP, albumin, lymphocyte count) quantifies early risk in older adults with dysphagia commencing tube feeding; a score obtained within 24–48 h should trigger enhanced surveillance and fast-track referral to multidisciplinary teams. Prospective, externally validated implementation trials—preferably leveraging electronic-health-record alerts—are required before clinical adoption. While several limitations of this study should be acknowledged. First, as a secondary analysis of a single-center retrospective study conducted in Japan, the results may be influenced by unmeasured confounders and inherent biases due to the retrospective design. Second, the moderate sample size (*n* = 253) was neither predetermined by a power calculation nor subjected to *post-hoc* power analysis, which may reduce statistical precision—especially in subgroup analyses—and increase the risk of false-positive findings. Third, the use of multivariable modeling carries a non-negligible risk of overfitting. Additionally, the generalizability of the findings is limited to Japanese patients; further validation in diverse ethnic and geographic populations is needed. Finally, given the exploratory nature of this *post hoc* analysis, further prospective studies are required to confirm these findings.

## Conclusion

The CALLY index, reflecting a patient's overall health status, serves as a valuable prognostic indicator for mortality and long-term outcomes in older adults with dysphagia.

## Data Availability

The original contributions presented in the study are included in the article/Supplementary material, further inquiries can be directed to the corresponding author.

## Supplementary material

Supplementary Figure 1 A density plot displaying the distribution of CALLY(A) and LN-CALLY(B) in the study. CALLY, C-reactive protein-albumin-lymphocyte index.

Supplementary Figure 2 Receiver-operating characteristic (ROC) curves comparing the 2-year mortality predictive performance of Ln-CALLY and the Prognostic Nutritional Index (PNI) in elderly patients with dysphagia receiving nutritional support.

Supplementary Figure 3 Sensitivity analysis showing the impact of different Ln-CALLY cut-off determination methods (median, original and tertile) on model performance.

Supplementary Figure 4 Comparison of Hazard Ratios for Ln-CALLY Using Various Imputation Techniques: Analysis of High vs. Low Ln-CALLY Groups.
