# Supplementary material for: Association between the C-reactive protein-albumin-lymphocyte (CALLY) index and mortality in elderly patients with dysphagia requiring nutritional support
Source: Front Neurol. 2025 Sep 30;16:1650795. doi: 10.3389/fneur.2025.1650795 (PMC12518111; doi:10.3389/fneur.2025.1650795)
Supplement: Supplementary Figure 1 — A density plot displaying the distribution of CALLY(A) and LN-CALLY(B) in the study. CALLY, C-reactive protein-albumin-lymphocyte index. [file Supplementary_file_1.zip › supplementary/supplementary figure 1.docx]

**Supplementary figure 1** A density plot displaying the distribution of CALLY(A) and LN-CALLY(B) in the study. CALLY, C-reactive protein-albumin-lymphocyte index.
